# Supplementary material for: Measures of retention in HIV care: A protocol for a mixed methods study
Source: PLoS One. 2024 Feb 23;19(2):e0294824. doi: 10.1371/journal.pone.0294824 (PMC10889855; doi:10.1371/journal.pone.0294824)
Supplement: S1 Appendix — (DOCX) [file pone.0294824.s001.docx]

**Measures of retention in HIV care: a mixed-method study**

**Qualitative interview guide**

| Date: |  | Interviewer: |  | Archival ID: |  |
| --- | --- | --- | --- | --- | --- |
| Start Time: |  | End Time: |  | Role in HIV care |  |
| Region of residence | |  |  |  |  |

**For interviewer**

1. Thank the interviewee for participating.
2. Introduce yourself.
3. Describe the purpose of the interview, benefits and potential harms, and permission to record.
4. State the duration of the interview.
5. Describe there will be monetary compensation.
6. Determine eligibility.

- 18 years or older
- Involved in HIV care.
- Speak English

Retention measure-related questions

1. How would you describe the term “retention in care” in your own words?
2. In our literature review, we found that people have used different measures for retention in care, we are now going to discuss some of these approaches with you.

What do you think about the following components of measuring retention in care:

- Follow-up time (3 months, 3-6 months, 6-12 months, 12 + months)
- Type of visits (clinical visits, pharmacy visits, laboratory visits, administrative records)
- Type of count (kept/missed visit counts, gap scores: gaps in care between completed visits based on predetermined time intervals)
- Scheduled visits (emergency or missed visits)
- Group-level definitions
- Composite definitions (single measure with two or three components)

1. Are you aware of any other approaches to measuring retention in care in addition to the ones listed above?
2. What do you think is the best way of measuring retention in care?
3. Will you prefer separate standards for different settings such as resource-rich and resource-constraint settings, research, and programs?
4. Any additional thoughts you would like to share?

Thank you for completing the interview with us. A collaborative effort in how to define and measure retention will help harmonize the care PLHIV provides across the globe. Your time and input are valuable for our study.
